# Can a Traditional Korean Manual Therapy Be a Complementary and Alternative Strategy for Cervicogenic Dizziness? A Study Protocol for a Randomized Controlled Trial

**DOI:** 10.1155/2018/1062593

**Published:** 2018-06-03

**Authors:** Seungwon Shin, Jinyoung Kim, Ami Yu, Hyung-Sik Seo, Mi-Ran Shin, Seung-Ug Hong, Chan Yung Jung, Koh-Woon Kim, Jae-Heung Cho, Euiju Lee

**Affiliations:** ^1^Department of Clinical Korean Medicine, Graduate School, Kyung Hee University, 26 Kyungheedae-ro, Dongdaemun-gu, Seoul 02447, Republic of Korea; ^2^STAT Department, HERINGS, 726 Nonhyeon-ro, Gangnam-gu, Seoul 06051, Republic of Korea; ^3^Department of Korean Medical Ophthalmology & Otolaryngology & Dermatology, School of Korean Medicine, Pusan National University, 20 Geumo-ro, Mulgeum-eup, Yangsan, Gyeongsangnam-do 50612, Republic of Korea; ^4^Department of Sasang Constitutional Medicine, College of Oriental Medicine, Semyung University, 65 Semyung-ro, Jecheon, Chungcheongbuk-do 27136, Republic of Korea; ^5^Department of Oriental Medicine Ophthalmology & Otolaryngology & Dermatology, College of Oriental Medicine, Dongguk University, 27 Dongguk-ro, Ilsandong-gu, Goyang, Gyeonggi-do 10326, Republic of Korea; ^6^Institute of Oriental Medicine, College of Korean Medicine, Dongguk University, 123 Dongdae-ro, Gyeongju-si, Gyeongsangbuk-do 38066, Republic of Korea; ^7^Department of Korean Rehabilitation Medicine, College of Korean Medicine, Kyung Hee University, 23 Kyungheedae-ro, Dongdaemun-gu, Seoul 02447, Republic of Korea; ^8^Department of Sasang Constitutional Medicine, College of Korean Medicine, Kyung Hee University, 23 Kyungheedae-ro, Dongdaemun-gu, Seoul 02447, Republic of Korea

## Abstract

Cervicogenic dizziness is dizziness triggered by movement or positioning of the cervical spine and is often accompanied by neck pain or stiffness. This is a prospective, pragmatic, assessor-blind, randomized controlled trial aimed at testing the efficacy and safety of adjuvant Chuna Manual Therapy (CMT) in patients with cervicogenic dizziness under usual care treatments. Fifty patients with cervicogenic dizziness will be randomly allocated to CMT or usual care (UC) groups in a 1 : 1 ratio. Extensive screening procedures, including examinations for central nervous system problems and nystagmus, will be applied to exclude other dizziness-inducing disorders. The eligible participants will receive 12 sessions of CMT plus UC or only UC over 6 weeks. CMT includes mandatory and discretionary techniques, whereas UC includes electrotherapy, thermotherapy, and patient education. The efficacy will be evaluated primarily as Dizziness Handicap Inventory score. The severity and frequency of dizziness, the level of neck pain or stiffness, and the cervical range of motion will also be evaluated. Safety will be assessed by adverse events. The data will be statistically analyzed at *p* < 0.05.* Trial Registration*. This trial was registered with Clinical Research Information Service (CRIS) in Korea, KCT0002565, on 29 November 2017, https://cris.nih.go.kr/cris/search/search_result_st01_kren.jsp?seq=9610&ltype=&rtype=.

## 1. Introduction

Cervicogenic dizziness, also known as cervical dizziness, is dizziness originating from the cervical spine [1], in other words, symptoms of giddiness triggered by cervical movements or positions, possibly accompanied by neck pain or stiffness [2]. The prevalence of cervicogenic dizziness is unclear; however, it has been reported that 65 to 66% of dizziness in the elderly could be attributed to cervical spine dysfunction, including spondylosis [2, 3], which is a common [4] and increasingly prevalent [5] cause of dizziness.

The causes of cervicogenic dizziness are controversial [6]. A recent review suggested that Barré-Liéou syndrome, proprioceptive cervical vertigo, bow-hunter syndrome, and migraine could induce cervicogenic dizziness, but this has not yet been confirmed [3]. Another review suggested that whiplash-associated disorders or degenerative cervical disorders could be causes [4]. Because of this uncertainty, physicians usually diagnose cervicogenic dizziness by exclusion [7].

The treatment of cervicogenic dizziness has not yet been standardized. Physical therapies [3, 5, 8], surgery [3, 7], nonsteroidal anti-inflammatory drugs (NSAIDs) [9], trigger point injection with lidocaine [5], acupuncture therapy [9], and patient education [10], as well as miscellaneous manual therapies [2, 11–14], have been explored in previous studies for reducing the severity and frequency of dizziness and improving the cervical range of motion, but no definite conclusions have been reached.

Chuna Manual Therapy (CMT) is a spinal manipulation technique based on the traditional Korean medicine (TKM) theory that both function and structure have systematic correlations. It is a therapeutic technique for modulating both orthopedic and functional balances simultaneously [15]. CMT usually involves high-velocity and low-amplitude thrusts to the joints and applies manual force to joints in the passive range of motion [16]. It is widely used in clinical practice for patients with musculoskeletal pain, such as low back pain, neck pain, or temporomandibular joint disorders [15]. A few clinical studies conducted in China have shown the efficacy of CMT or similar manual therapies for cervicogenic dizziness or vertigo. CMT has been compared with nimodipine administration [17], traction therapy [18], or combined treatment with acupuncture [19, 20], using randomized or nonrandomized designs. However, most of these interventions were carried out using muscle or tissue massage, which are not the similar techniques to Korean CMT spinal manipulation. Moreover, most of these studies assessed efficacy using invalidated response measures. Given the overall low quality of the studies and the differences in CMT techniques, there remains a need to rigorously demonstrate a clinical effect of CMT on cervicogenic dizziness.

The objective of this randomized controlled trial (RCT) is to test the efficacy and safety of 6-week CMT in male or female subjects, aged between 20 and 70 years, with cervicogenic dizziness, and compare them with the results from usual care (UC).

## 2. Materials and Methods

The trial title is “Adjuvant Chuna Manual Therapy in Subjects with Cervicogenic Dizziness (CHERIE Study): A Study Protocol for a Prospective, Pragmatic, Assessor-Blind, Randomized Controlled Trial.” This study protocol was developed in accordance with the Standard Protocol Items: Recommendations for Interventional Trials (SPIRIT) 2013 statement [21]. Because there are no CMT-specific recommendations for trial design, we referred instead to the Revised Standards for Reporting Interventions in Clinical Trials of Acupuncture (STRICTA): extending the CONSORT statement [22]. The relevant SPIRIT checklist is attached as* supplementary data* (available here).

### 2.1. Objectives and Hypothesis

This study is aimed primarily at exploring the therapeutic effect of adjuvant CMT for 6 weeks in male and female patients (20–70 years old) with cervicogenic dizziness, previously under usual care treatments. The null hypothesis is that the mean Dizziness Handicap Inventory (DHI) scores at baseline and at week 6 will be equal for the CMT and UC groups.

Second, the severity and frequency of cervicogenic dizziness after CMT or UC will be evaluated, along with other outcome measures. Differences between the two groups in neck pain, stress level, and quality of life (QoL) will also be assessed. The safety of CMT will be examined using adverse event (AE) reporting.

### 2.2. Study Design

**Figure 1:**
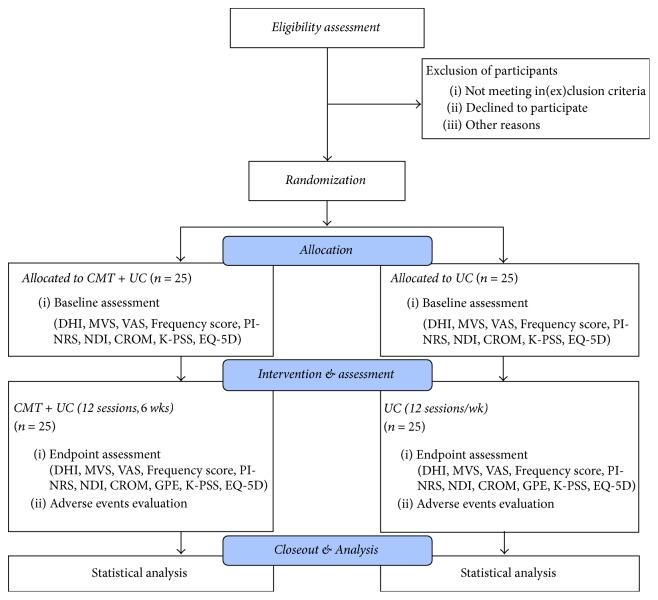
*Study flowchart*. CMT, Chuna Manual Therapy; CROM, cervical range of motion; DHI, Dizziness Handicap Inventory; EQ-5D, EuroQol Five-Dimension Questionnaire; GPE, global perceived effect; K-PSS, Korean version of Perceived Stress Scale; MVS, mean vertigo scale; NDI, neck disability index; PI-NRS, Pain Intensity Numerical Rating Scale; UC, usual care; VAS, visual analog scale.

This study is a prospective, pragmatic, assessor-blind, randomized controlled trial. Fifty patients complaining of recurring dizziness with cervical origins, with DHI score ≥ 16 at baseline, will be randomly allocated to either the CMT or UC group, with a 1 : 1 ratio. After eligibility assessment and a 1-week run-in period, the participants will receive 12 sessions of CMT combined with UC, or UC only, over 6 weeks. CMT consists of mandatory and discretionary techniques, and skilled KMD doctors will perform the techniques considered to be optimal for each patient's condition. This latitude in treatment selection is why the study is designated as pragmatic. UC includes electrotherapy, thermotherapy, and patient education. A flowchart and timetable for the study are shown in Figure 1 and Table 1, respectively.

### 2.3. Setting and Recruitment

Two TKM hospitals will recruit eligible patients: the Kyung Hee University Korean Medicine Hospitals in Hoegi and Gangdong in Seoul, Republic of Korea. Participants will be recruited mainly from outpatient clinics. Recruiting advertisements will be posted on hospital web pages and noticeboards.

### 2.4. Ethical Review and Trial Registration

The study protocol (version 1.0) and informed consent form were peer-reviewed and approved by the Institutional Review Board of Kyung Hee University Korean Medicine Hospital on 11 August 2017 (KOMCIRB-170717-HR-026), in accordance with scientific and ethical regulations, that is, Good Clinical Practice and relevant laws of the Ministry of Food and Drug Safety in Korea. The trial was registered at CinicalTrials.gov on 19 September 2017 (NCT03291912, https://clinicaltrials.gov/show/NCT03291912). Personal information will not be collected and only encrypted code will be used for trial records.

### 2.5. Eligibility Criteria

The inclusion criteria are as follows:

1. Men or women aged between 20 and 70 years
2. Neck pain or stiffness with dizziness related to movement or positioning of the cervical spine
3. Recurring symptoms of dizziness for 1 month or more
4. DHI ≥ 16 at baseline
5. Informed consent for study participation

The exclusion criteria are as follows:

1. Dizziness induced by vestibular disorders (benign paroxysmal positional vertigo, peripheral vestibulopathy, Meniere disease, vestibular neuritis, etc.)
2. Dizziness induced by central nervous system (CNS) disorders (cerebellar ataxia, cerebellar infarction/hemorrhage, demyelination, vertebrobasilar insufficiency, epilepsy, increased intracranial pressure, Parkinson's disease, migraine, etc.)
3. Dizziness induced by cardiovascular disorders (arrhythmia, heart valve disease, anemia, orthostatic hypotension, coronary artery disease, etc.)
4. Dizziness induced by active or uncontrolled disease (uncontrolled diabetes mellitus, hypertension, respiratory or endocrinological disorders, etc.)
5. Drug-induced dizziness
6. Severe chronic or terminal diseases (malignant cancer, tuberculosis, etc.)
7. Chronic psychiatric diseases under treatment (depression, panic disorder, etc.)
8. Medical conditions for which CMT is forbidden (spinal tumor, acute fracture, infectious spondylopathy, congenital malformations of the spine, history of spinal operation within the past 3 months, progressive neurological damage, severe neurological symptoms, spinal fixation devices, syringomyelia, hydrocephaly, etc.)
9. Treatment within the previous 1 week for cervicogenic dizziness (NSAIDs, steroid, herbal drug, acupuncture, manual therapy, etc.)
10. Women with (suspected) pregnancy or breast-feeding
11. Suspicion of alcohol or drug abuse
12. Participation in another clinical study within the previous 1 month
13. Difficulty in communicating with the investigators
14. Other unanticipated reasons for ineligibility to participate

### 2.6. Dropout Criteria

Enrolled participants will be dropped if (a) the investigators detect ineligibility after random allocation, (b) participants withdraw consent for participation, refuse to receive CMT or UC, or are lost to follow-up, (c) serious adverse events or complications that need treatment are reported, or (d) the participants or investigators significantly violate the study protocol, including the criteria concerning concomitant medication.

### 2.7. Randomization, Allocation, and Blinding

Random number sequences will be generated by an independent statistician (Ami Yu) using SAS® 9.4 software (SAS Institute Inc., Cary, NC). The randomization will be block-stratified by the recruitment hospital to keep the numbers of each group similar throughout the study. The statistician will provide each hospital with sealed envelopes, in each of which a random number and an allocated group are written. Immediately after a participant is found to be eligible according to the screening process, a TKM doctor who will administer UC or CMT will open the next envelope in the sequence. Because this study is open-labeled to the investigators and patients, only the outcome assessors will be blinded. The outcome assessors will be TKM doctors different from those who administer CMT and UC. Only clinical emergency situations will allow breaking of the randomization code and blinding. At the end of the study, each outcome assessor will be asked which group he or she thought the patient was allocated to, and the answers will be used to calculate a blinding index (BI) [23].

### 2.8. Chuna Manual Therapy (CMT)

The CMT regimen for cervicogenic dizziness has been determined based on a textbook for Chuna manual medicine [24], several previous CMT studies for neck pain [25–29], and experts' opinions. Since this pragmatic study was designed to reflect clinical settings, the CMT techniques are individualized depending on each patient's condition. We also referred to a lower back pain study with similar CMT performance [16]. The final regimen was externally reviewed by the Korean Society of Chuna Manual Medicine for Spine & Nerve (http://www.chuna.or.kr), and the feasibility of the approach for cervicogenic dizziness was confirmed.

The CMT techniques in this study consist of mandatory and discretionary parts. The mandatory techniques are flexion distraction and dysfunction correction for the cervical spine, while the discretionary techniques apply to other regions (thoracic or lumbar parts). Only TKM doctors who have completed the regular CMT curriculum and have had 1 year or more of experience in clinical practice will be qualified as CMT practitioners for this study. The practitioners will assess the patients before each session and decide which discretionary techniques are necessary for each patient. The techniques applied will be reported for every session, using case report forms. The detailed CMT regimen is shown in Table 2. The procedures that will be followed are derived from an accredited textbook [24].

Participants who are allocated to the CMT group will receive 12 sessions of CMT over 6 weeks (2 sessions/week).

### 2.9. Usual Care (UC)

No standard therapy for cervicogenic dizziness has been determined yet. A review study suggested that cervicogenic dizziness should be managed in the same way as neck pain [3]. Therefore, we have decided to educate the patients and allow both groups to have TKM-based physical therapies for neck symptoms, based on previous studies [3, 5, 8, 10] and clinical opinions. For the purposes of this study, these treatments are designated as UC.

The patient education program will include (a) physical and pathological information concerning cervicogenic dizziness, (b) causes and risk factors for cervicogenic dizziness, (c) muscle functions related to cervicogenic dizziness, and (d) home exercising skills for management of cervicogenic dizziness. Physical therapy consists of electrical stimulation (either meridian muscle interferential current electricity or meridian transcutaneous electricity) and heat stimulation (either a heat pack or an infrared lamp) to relieve pain or stiffness in the neck muscles.

All participants who are allocated to the CMT or UC groups will receive 12 sessions of UC for 6 weeks (2 sessions/week).

### 2.10. Concomitant Medications

Drug administration (betahistine, difenidol, flunarizine, cinnarizine, dimenhydrinate, ginko, meclizine, metoclopramide, scopolamine, pentoxifylline, perphenazine, ergoloid mesylate, droperidol, phenobarbital, prochlorperazine, promethazine, trimethobenzamide, vertigoheel, NSAIDs, steroids, etc.), herbal remedies, manual therapies, any type of acupuncture, moxibustion, or cupping, to relieve dizziness, back pain, or stiffness, will be forbidden throughout the study.

Any signs of the use of drugs that can induce dizziness will be monitored closely during every visit.

### 2.11. Screening Process

After informed consent is obtained, each patient will undergo a screening process for eligibility assessment. Demographic information (initial of name, age, and sex), physical examination results (height, weight, and body mass index), vital signs (blood pressure, pulse, and body temperature), and pregnancy test results (if available) will be collected. The medical history (past and present) and concomitant medications or therapies will be investigated. To exclude other types of dizziness, we will also administer tests of CNS function (deep tendon reflex, Hoffman sign, Babinski sign, ankle clonus, finger-to-nose, finger-to-finger, rapid alternating movement, heel-to-shin, Romberg's test, Brudzinski's sign, Kernig's sign, Naffziger's test, and Gorge's test) and nystagmus tests (spontaneous, gaze-evoked, positional, and positioning (Dix-Hallpike) nystagmus tests). If a patient does not seem to have any other identifiable cause of dizziness, the final decision on eligibility will be made by a TKM doctor.

### 2.12. Outcomes

#### 2.12.1. Primary Outcome

*Dizziness Handicap Inventory (DHI). *The DHI is a patient-rated outcome (PRO) assessment that evaluates dizziness-induced impairments functionally, emotionally, and physically [30]. A Korean version of the scale has been validated [31]. The scale, consisting of 25 items with 4 points for each (scores 0–100), is frequently used for cervicogenic dizziness [2, 10–13]. The primary endpoint is defined as the mean difference of DHI total scores between day 0 and week 6. Subordinately, the intergroup effect at an intermediate time point (the mean difference between day 0 and week 3), as well as intragroup effects within each group, will be analyzed.

#### 2.12.2. Secondary Outcomes

The following outcomes will be evaluated at day 0, week 3, and week 6. The mean differences of scores for each scale (week 3–day 0; week 6–day 0) will be compared between the CMT and UC groups. Intragroup effects will also be explored for each outcome measurement.

*Mean Vertigo Score (MVS).* The MVS is a PRO assessing the intensity of dizziness [32–39], consisting of 12 items, each with 0 (none) to 4 (very strong) options. The score is the arithmetic mean of the total sum (range: 0–4).

*Visual Analog Scale (VAS). *The VAS will be measured as in previous studies of cervicogenic dizziness [2, 8, 9, 11–13] to rate the severity of dizziness. The zero end of the line represents “not dizzy at all,” whereas the other end (scored as 10 points) represents “the dizziest I can imagine being.”

*Dizziness Frequency. *Patients will be asked how many episodes of dizziness they experienced since the last assessment. The frequency will be scored as 0 (none), 1 (less than once per month), 2 (1–4 episodes per month), 3 (1–4 episodes per week), 4 (once per day), or 5 (twice or more per day), as in previous studies [11–13].

*Pain Intensity Numerical Rating Scale (PI-NRS). *The PI-NRS is an 11-point Likert scale that assesses the severity of neck pain perceived by a patient. This scale is widely used for pain studies [40], including neck pain [41]. A patient is asked to pick one value from 0 (not painful) to 10 (most painful) based on the pain level for the past 24 hours. Because cervicogenic dizziness often coincides with neck pain or stiffness, this scale has been included in this study.

*Neck Disability Index (NDI). *The NDI is a questionnaire that measures the level of disability of the cervical spine, including 0 to 5 points for each of 10 items: pain, personal care, lifting, reading, headaches, concentration, work, driving, sleeping, and recreation [42]. The final NDI score is interpreted as the level of disability (no, mild, moderate, severe, or complete disability). We plan to use a validated Korean version of the NDI in this study [43]. A recent clinical trial for cervicogenic dizziness also used the NDI for efficacy assessment [10].

*Cervical Range of Motion (CROM). *The CROM has been measured in many clinical studies of cervicogenic dizziness [2, 9–12]. Outcome assessors will evaluate the CROM for the cervical spine of a patient in flexion, extension, lateral bending, and rotation.

*Global Perceived Effect (GPE). *The GPE is a validated scale evaluating a patient's perception of symptom worsening or improvement after an intervention [44]. It consists of 7 options (1 for completely recovered to 7 for worst ever). Several studies of cervicogenic dizziness have used this assessment at the end of the intervention [2, 11–13].

*Korean Version of Perceived Stress Scale (K-PSS).* The K-PSS is the Korean version of the PSS, which measures the perceived level of stress for the previous 1 month [45]. It has 10 questions, and each can be answered with 1 (never), 2 (almost never), 3 (sometimes), 4 (fairly often), or 5 (very often).

*EuroQol Five-Dimension Questionnaire (EQ-5D). *The EQ-5D will be used to assess quality of life, consisting of two parts (EQ-5D-5L and EQ-VAS). EQ-5D-5L has 5 dimensions (mobility, self-care, usual activities, pain/discomfort, and anxiety/depression) and each dimension has 5 levels (no problems, slight problems, moderate problems, severe problems, and extreme problems) [46]. EQ-VAS rates a patient's health on a 20 cm vertical visual analog scale, with endpoints labelled “the best health you can imagine” and “the worst health you can imagine.” The EQ-5D has been translated into Korean and validated [47].

### 2.13. Blinding Assessment

This is an open-label study, but the outcome assessors will be blinded throughout. After the final assessment of each participant, the assessors will be asked which group they thought the patient belonged to: the CMT group, the UC group, or do not know. The blinding index (BI) will be calculated as described in Bang's study [23]. The index ranges from 1 (complete lack of blinding) to 0 (consistent with perfect blinding) to −1 (always guessed incorrectly). If the confidence interval (CI) of the estimated BI includes zero, the blinding will be considered successful.

### 2.14. Safety Assessment

AEs will be reported for every visit during the treatment period. The intensity and frequency of each event will be monitored. If serious AEs occur, postmanagement will be properly conducted. Local discomfort or pain, headache, fatigue, or referred pain can occur immediately after CMT but are usually mild and temporary.

### 2.15. Sample Size Calculation

RCTs evaluating the efficacy of a manual therapy, compared with placebo, for cervicogenic dizziness gave values for the mean changes and standard deviations [11, 13]. With 15.9 as the mean difference between the intervention and control groups and 12.91 as the standard deviation, the necessary sample size calculated using PASS 14 was 12 per group (two-sided, *α* = 0.05 and 1 − *β* = 0.9). We will allocate a total of 50 participants (25 per group), allowing for an anticipated drop-out rate of 40%.

### 2.16. Statistical Analysis

The full analysis set is defined as the group of participants who received CMT or UC and were assessed for the primary outcome at least once. The “per protocol” set is defined as the group of participants who completed all planned interventions and outcome assessments. Efficacy data will be analyzed primarily using the full analysis set and secondarily using the per protocol set. The missing data will be imputed using the “last observation carried forward” method. Safety data will be analyzed using the safety assessment set, which is defined as the group of participants who received CMT or UC at least once.

The primary and secondary outcomes will be statistically analyzed using two-sample *t*-tests or Wilcoxon's rank-sum tests for intergroup comparisons and paired *t*-tests or Wilcoxon's signed-rank tests for intragroup comparisons. Categorical variables from demographic data or AEs will be analyzed using the Chi-square test or Fisher's exact test.

An independent statistician (Ami Yu) will analyze the data at *p* < 0.05 with SAS 9.4 software (SAS Institute Inc.).

## 3. Results and Discussion

This study protocol is for a prospective, pragmatic, assessor-blind, randomized controlled trial to test the efficacy and safety of TKM-based CMT in subjects with cervicogenic dizziness. Fifty participants will be randomly allocated to CMT or UC groups. Over 6 weeks, the CMT group will undergo 12 sessions of CMT, physical therapy, and patient education, whereas the UC group will undergo the same number of sessions of physical therapy and patient education. The primary outcome will be assessed as the change in a validated PRO, the DHI, between the baseline and closing time point. This is expected to be the first randomized controlled trial in Korea to perform CMT for cervicogenic dizziness. It is also meaningful to adopt CMT for neurological disorders, not just for musculoskeletal symptoms.

There are no CMT-specific recommendations for trial design, and few clinical trials of high quality have been published. We therefore followed the recommendations for acupuncture studies, or STRICTA [22], as much as possible. Because the CMT regimen consists of mandatory and discretionary techniques and the discretionary ones depend on the individual's condition, we strictly defined the qualifications required of CMT practitioners for this study and plan to document all CMT treatment decisions.

Another challenge was the screening process to identify eligible participants. As mentioned above, to diagnose cervicogenic dizziness, physicians must rule out other relevant disorders that can trigger dizziness [7]. Dizziness can arise not only from a variety of central or peripheral diseases but also from drug-related side effects. This was why we organized a research team with medical experts from various areas, including neurologists (Euiju Lee and Mi-Ran Shin), ophthalmologists (Seung-Ug Hong and Hyung-Sik Seo), physiatrists (Jae-Heung Cho and Koh-Woon Kim), and a medical methodologist (Seungwon Shin). We intend to perform full examinations of the CNS and nystagmus, as well as extensive physical examinations and check-ups for medical history and concomitant medications. Cooperation and communication among the research teammates will be an essential factor toward a successful study.

## 4. Conclusion

We expect that this randomized controlled trial can provide a new complementary and alternative strategy to manage cervicogenic dizziness with adjuvant traditional manual therapy or CMT.

## Figures and Tables

**Table 1:** Study timetable.

	Screening	Randomization	Treatment							
Visits	V1	V2	V3	V4-V5	V6	V7	V8-V9	V10-V11	V12	V13
Time point	*−1 W*	*Day 0*	*1 W ± 2 D*	*2 W ± 2 D*	*3 W ± 2 D*		*4 W ± 2 D*	*5 W ± 2 D*	*6 W ± 2 D*	
**Enrollment:**										
*Informed consent*	×									
*Demographics *	×									
*Physical examination*	×									
*Vital signs*	×									
*Pregnancy test*	×									
*Medical history*	×									
*Concomitant medication*	×	×	×	×	×	×	×	×	×	×
*CNS examination*	×									
*Nystagmus examination*	×									
*Eligibility assessment*	×									
*Random allocation*		×								

**Interventions:**										
*CMT (twice/week)*		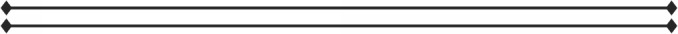								
*UC (twice/week)*										

**Assessments:**										
*DHI*	×	×				×				×
*MVS*		×				×				×
*VAS*		×				×				×
*Frequency*		×				×				×
*PI-NRS*		×				×				×
*NDI*		×				×				×
*CROM*		×				×				×
*GPE*										×
*K-PSS*		×				×				×
*EQ-5D*		×				×				×
*New BI*										×
*Adverse events*		×	×	×	×	×	×	×	×	×

BI, blinding index; CMT, Chuna Manual Therapy; CNS, central nervous system; CROM, cervical range of motion; DHI, Dizziness Handicap Inventory; EQ-5D, EuroQol Five-Dimension Questionnaire; GPE, global perceived effect; K-PSS, Korean version of Perceived Stress Scale; MVS, mean vertigo scale; NDI, neck disability index; PI-NRS, Pain Intensity Numerical Rating Scale; UC, usual care; V, visit; VAS, visual analog scale; W, week.

**Table 2:** CMT regimen for cervicogenic dizziness.

Regions	CMT techniques
Cervical spine	(i) Supine cervical distraction technique^*∗*^
	(ii) Supine cervical dysfunction correction technique^*∗*^
	(iii) Supine cervical dysfunction JS distraction/correction technique^*∗*^
	(iv) Prone cervical distraction technique

Thoracic spine	(i) Sitting upper thoracic dysfunction muscle release/strengthening technique (extension or flexion)
	(ii) Supine thoracic extension dysfunction correction technique
	(iii) Prone lower thoracic flexion dysfunction correction technique
	(iii) Sitting lower thoracic dysfunction muscle release/strengthening technique (extension or flexion)

Lumbar spine	(i) Prone lumbosacral distraction technique
	(ii) Sidelying lumbar rocking distraction technique
	(iii) Sidelying lumbar dysfunction correction technique (extension or flexion)
	(iv) Sitting lumbar flexion dysfunction muscle release/strengthening technique
	(v) Spine flexion distraction technique (flexion, extension, circumduction, sidebending, and magnum pump)

*Note*. The techniques marked by asterisks are mandatory, whereas the others are discretionary. The practitioners will evaluate each patient before the CMT sessions and perform individually necessary techniques to improve cervicogenic dizziness. CMT, Chuna Manual Therapy.

## Data Availability

The datasets generated and analyzed in this study will be available from the corresponding author upon reasonable request.

## Abbreviations

AE: Adverse event
CI: Confidence interval
CMT: Chuna Manual Therapy
DHI: Dizziness Handicap Inventory
UC: Usual care
MVS: Mean vertigo scale
VAS: Visual analog scale
PI-NRS: Pain Intensity Numerical Rating Scale
NDI: Neck disability index
CROM: Cervical range of motion
K-PSS: Korean version of Perceived Stress Scale
EQ-5D: EuroQol Five-Dimension Questionnaire
GPE: Global perceived effect
BI: Blinding index
TKM: Traditional Korean medicine
SPIRIT: Standard Protocol Items Recommendations for Interventional Trials
STRICTA: Standards for Reporting Interventions in Clinical Trials of Acupuncture
CNS: Central nervous system
NSAID: Nonsteroidal anti-inflammatory drug
PRO: Patient-rated outcome
QoL: Quality of life
RCT: Randomized controlled trial.

## Supplementary Materials

Supplementary Materials Standard Protocol Items: Recommendations for Interventional Trials (SPIRIT) checklist is attached as supplementary data.
