# Supplementary material for: Can a Traditional Korean Manual Therapy Be a Complementary and Alternative Strategy for Cervicogenic Dizziness? A Study Protocol for a Randomized Controlled Trial
Source: Evid Based Complement Alternat Med. 2018 Jun 3;2018:1062593. doi: 10.1155/2018/1062593 (PMC6008796; doi:10.1155/2018/1062593)
Supplement: Supplementary Materials — Standard Protocol Items: Recommendations for Interventional Trials (SPIRIT) checklist is attached as supplementary data. [file 1062593.f1.docx]

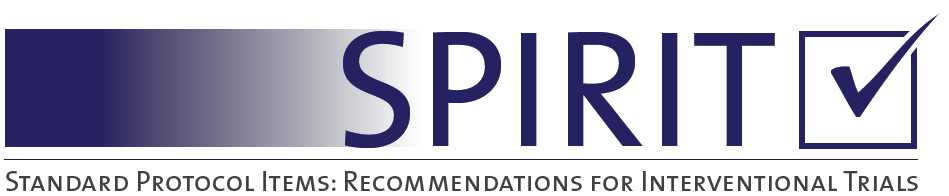


SPIRIT 2013 Checklist: Recommended items to address in a clinical trial protocol and related documents*

## Section/item Item No

**Description Addressed on**

**page number**

**Administrative information**

Title, 7-8

Title 1 Descriptive title identifying the study design, population, interventions, and, if applicable, trial acronym

# Abstract, 8

Trial registration 2a Trial identifier and registry name. If not yet registered, name of intended registry

# NA

2b All items from the World Health Organization Trial Registration Data Set

# 8

Protocol version 3 Date and version identifier

Funding 4 Sources and types of financial, material, and other support

# 22

22-23

Roles and responsibilities

5a Names, affiliations, and roles of protocol contributors

# 22

5b Name and contact information for the trial sponsor

5c Role of study sponsor and funders, if any, in study design; collection, management, analysis, and NA

interpretation of data; writing of the report; and the decision to submit the report for publication, including whether they will have ultimate authority over any of these activities

# NA

5d Composition, roles, and responsibilities of the coordinating centre, steering committee, endpoint

adjudication committee, data management team, and other individuals or groups overseeing the trial, if applicable (see Item 21a for data monitoring committee)

## Introduction

Background and rationale

# 5-6

6a Description of research question and justification for undertaking the trial, including summary of relevant

studies (published and unpublished) examining benefits and harms for each intervention

6b Explanation for choice of comparators Objectives 7 Specific objectives or hypotheses

Trial design 8 Description of trial design including type of trial (eg, parallel group, crossover, factorial, single group),

12

# 7

7-8

allocation ratio, and framework (eg, superiority, equivalence, noninferiority, exploratory)

## Methods: Participants, interventions, and outcomes

8

Study setting 9 Description of study settings (eg, community clinic, academic hospital) and list of countries where data will

be collected. Reference to where list of study sites can be obtained

# 8-10

Eligibility criteria 10 Inclusion and exclusion criteria for participants. If applicable, eligibility criteria for study centres and

individuals who will perform the interventions (eg, surgeons, psychotherapists)

# 11-13

Interventions 11a Interventions for each group with sufficient detail to allow replication, including how and when they will be

administered

# 10

11b Criteria for discontinuing or modifying allocated interventions for a given trial participant (eg, drug dose

change in response to harms, participant request, or improving/worsening disease)

# 11

11c Strategies to improve adherence to intervention protocols, and any procedures for monitoring adherence

(eg, drug tablet return, laboratory tests)

# 13

11d Relevant concomitant care and interventions that are permitted or prohibited during the trial

Outcomes 12 Primary, secondary, and other outcomes, including the specific measurement variable (eg, systolic blood

# 14-17

pressure), analysis metric (eg, change from baseline, final value, time to event), method of aggregation (eg, median, proportion), and time point for each outcome. Explanation of the clinical relevance of chosen

efficacy and harm outcomes is strongly recommended

# Table 1

Participant timeline 13 Time schedule of enrolment, interventions (including any run-ins and washouts), assessments, and visits for participants. A schematic diagram is highly recommended (see Figure)

# 17-18

Sample size 14 Estimated number of participants needed to achieve study objectives and how it was determined, including

clinical and statistical assumptions supporting any sample size calculations

# 8

Recruitment 15 Strategies for achieving adequate participant enrolment to reach target sample size

## Methods: Assignment of interventions (for controlled trials)

Allocation:

# 11

Sequence generation

16a Method of generating the allocation sequence (eg, computer-generated random numbers), and list of any factors for stratification. To reduce predictability of a random sequence, details of any planned restriction

(eg, blocking) should be provided in a separate document that is unavailable to those who enrol participants

or assign interventions

Allocation concealment mechanism

11

16b Mechanism of implementing the allocation sequence (eg, central telephone; sequentially numbered, opaque, sealed envelopes), describing any steps to conceal the sequence until interventions are assigned

# 11

Implementation 16c Who will generate the allocation sequence, who will enrol participants, and who will assign participants to

interventions

# 11

Blinding (masking) 17a Who will be blinded after assignment to interventions (eg, trial participants, care providers, outcome

assessors, data analysts), and how

# 11

17b If blinded, circumstances under which unblinding is permissible, and procedure for revealing a participant’s allocated intervention during the trial

## Methods: Data collection, management, and analysis

14-17

Data collection methods

18a Plans for assessment and collection of outcome, baseline, and other trial data, including any related processes to promote data quality (eg, duplicate measurements, training of assessors) and a description of

study instruments (eg, questionnaires, laboratory tests) along with their reliability and validity, if known.

Reference to where data collection forms can be found, if not in the protocol

# 10

18b Plans to promote participant retention and complete follow-up, including list of any outcome data to be collected for participants who discontinue or deviate from intervention protocols

# NA

Data management 19 Plans for data entry, coding, security, and storage, including any related processes to promote data quality (eg, double data entry; range checks for data values). Reference to where details of data management

procedures can be found, if not in the protocol

# 18-19

Statistical methods 20a Statistical methods for analysing primary and secondary outcomes. Reference to where other details of the

statistical analysis plan can be found, if not in the protocol

# NA

20b Methods for any additional analyses (eg, subgroup and adjusted analyses)

20c Definition of analysis population relating to protocol non-adherence (eg, as randomised analysis), and any 18

statistical methods to handle missing data (eg, multiple imputation)

## Methods: Monitoring

NA

Data monitoring 21a Composition of data monitoring committee (DMC); summary of its role and reporting structure; statement of whether it is independent from the sponsor and competing interests; and reference to where further details

about its charter can be found, if not in the protocol. Alternatively, an explanation of why a DMC is not

needed

# NA

21b Description of any interim analyses and stopping guidelines, including who will have access to these interim

results and make the final decision to terminate the trial

# 17

Harms 22 Plans for collecting, assessing, reporting, and managing solicited and spontaneously reported adverse

events and other unintended effects of trial interventions or trial conduct

# NA

Auditing 23 Frequency and procedures for auditing trial conduct, if any, and whether the process will be independent from investigators and the sponsor

## Ethics and dissemination

8

Research ethics approval

Protocol amendments

1. Plans for seeking research ethics committee/institutional review board (REC/IRB) approval

# 21(Trial status

1. Plans for communicating important protocol modifications (eg, changes to eligibility criteria, outcomes, analyses) to relevant parties (eg, investigators, REC/IRBs, trial participants, trial registries, journals,

regulators)

# 8

Consent or assent 26a Who will obtain informed consent or assent from potential trial participants or authorised surrogates, and

how (see Item 32)

# NA

26b Additional consent provisions for collection and use of participant data and biological specimens in ancillary

studies, if applicable

# 8

Confidentiality 27 How personal information about potential and enrolled participants will be collected, shared, and maintained

in order to protect confidentiality before, during, and after the trial

Declaration of interests

22

28 Financial and other competing interests for principal investigators for the overall trial and each study site

# 22

Access to data 29 Statement of who will have access to the final trial dataset, and disclosure of contractual agreements that

limit such access for investigators

Ancillary and post- trial care

NA

30 Provisions, if any, for ancillary and post-trial care, and for compensation to those who suffer harm from trial

participation

# NA

Dissemination policy 31a Plans for investigators and sponsor to communicate trial results to participants, healthcare professionals, the public, and other relevant groups (eg, via publication, reporting in results databases, or other data

sharing arrangements), including any publication restrictions

# NA

31b Authorship eligibility guidelines and any intended use of professional writers

# 22

31c Plans, if any, for granting public access to the full protocol, participant-level dataset, and statistical code

## Appendices

Informed consent materials

Biological specimens

# NA

1. Model consent form and other related documentation given to participants and authorised surrogates

# NA

1. Plans for collection, laboratory evaluation, and storage of biological specimens for genetic or molecular analysis in the current trial and for future use in ancillary studies, if applicable

*It is strongly recommended that this checklist be read in conjunction with the SPIRIT 2013 Explanation & Elaboration for important clarification on the items. Amendments to the protocol should be tracked and dated. The SPIRIT checklist is copyrighted by the SPIRIT Group under the Creative Commons “Attribution-NonCommercial-NoDerivs 3.0 Unported” license.
